# A pilot project to increase health literacy among youth from seasonal farmworker families in rural eastern North Carolina: a qualitative exploration of implementation and impact

**DOI:** 10.5195/jmla.2019.560

**Published:** 2019-04-01

**Authors:** Israel M. Mendez, Mary Lisa Pories, Leah Cordova, Andreina Malki, Melinda F. Wiggins, Joseph G. L. Lee

**Affiliations:** Research Assistant, Department of Health Education and Promotion, College of Health and Human Performance, East Carolina University, Greenville, NC, mendezi14@students.ecu.edu; College of Health and Human Performance, East Carolina University, Greenville, NC, poriesm@ecu.edu; STEM Librarian, Research and Instructional Services, Joyner Library, East Carolina University, Greenville, NC, lcordova@uthsc.edu; Youth Director, Student Action with Farmworkers, Durham, NC, andreina@saf-unite.org; Executive Director, Student Action with Farmworkers, Durham, NC, mwiggins@duke.edu; Department of Health Education and Promotion, College of Health and Human Performance, and Center for Health Disparities, Brody School of Medicine, East Carolina University, Greenville, NC, leejose14@ecu.edu

## Abstract

**Objective:**

There are substantial health inequalities for seasonal agricultural workers and their families in the United States. One identified inequality is in health literacy. The authors explored the implementation and impact of connecting youth from seasonal farmworker families who participated in a leadership and college pipeline program with Internet access by providing a tablet with a paid cellular data plan and university library–based health literacy training.

**Methods:**

With the support of a National Network of Libraries of Medicine Health Information Outreach Award, we conducted a qualitative, utilization-focused evaluation by conducting semi-structured interviews from December 2017 through February 2018 with middle and high school age participants in the program (n=10). After parental consent and youth assent, we recorded interviews with participants at program activity locations or in their homes. We then utilized inductive thematic analysis with 2 primary coders.

**Results:**

We identified four themes: (1) having access to the Internet can be transformative, (2) access resulted in increased knowledge of and interest in one’s own and others’ health, (3) “Google” is the norm, and (4) participant training increased self-efficacy to determine credible sources and resources.

**Conclusion:**

Providing Internet access and iPads was possible to implement and resulted in increased utilization of health information. The combination of Internet access with training on information literacy was a key factor in achieving these positive outcomes. The findings suggest the importance of ensuring equitable access to the Internet in efforts to improve educational and health outcomes for seasonal farmworkers and their families.

## INTRODUCTION

Political, legal, and economic forces have limited the pay, labor organizing abilities, and upward social mobility of migrant and seasonal farmworkers [1]. Profound issues of social injustice make migrant and seasonal agricultural work one of the most dangerous occupations in the United States [2, 3]. Between 1992 and 2006, heat stroke killed 7 farmworkers in North Carolina (NC) [4]. One survey found that almost 80% of NC farmworkers had skin diseases [5]. More than a quarter of children in farmworker families had not received dental care in a survey in NC and Virginia [6]. Pesticide exposure in farmworker children was documented as a common occurrence [7]. Over half of children of migrant farmworkers had unmet medical needs in one survey [8]. Researchers found substantial barriers to health care [9]. Farm housing quality standards are low, inspections are limited [10], and indoor temperatures can be sweltering during the summer [11].

Among other structural barriers that hinder health, access to evidence-based consumer health information is limited by geography, language, and lack of Internet connectivity [2, 12–14]. For example, inadequate access to information is considered a contributing factor for higher rates of HIV and other sexually transmitted infections among migrant and seasonal farmworkers [2]. Access to high-quality health information is a critical part of health literacy. Health literacy is defined as including “the degree to which individuals have the capacity to obtain, process, and understand basic health information and services needed to make appropriate health decisions” [15]. Thus, attention to health literacy is a necessary part of addressing health inequalities for migrant and seasonal farmworkers and their families.

This project sought to enhance the infrastructure of a farmworker advocacy organization’s youth leadership development and educational access program by providing iPads, Internet connectivity, and university library–based health information literacy training to middle and high school youth from participating farmworker families. The aim of this study was to explore the implementation and impacts of this pilot effort.

## METHODS

### Research design

The research design was inspired by principles of “utilization-focused evaluation,” in which the purpose of the research is to provide useful information to the designers and implementers of the program [16]. Thus, the focus is less on topics that typically interest researchers (e.g., proving a theory or formally testing an intervention’s efficacy) and is instead centered on providing useful information to inform the program being evaluated. To that end, the authors used a posttest only design with qualitative interviews to capture themes about implementation and impact.

### Setting

This project was a partnership between the East Carolina University (ECU) Joyner Library, the ECU Department of Health Education and Promotion, and Student Action with Farmworkers (SAF). Funding came from a National Network of Libraries of Medicine Health Information Outreach Award. Author Lee, a faculty member in public health who had been a migrant health outreach intern with SAF in 2004, approached author Cordova, a departmental library liaison, and SAF about collaborating on the project.

SAF has operated the Levante Leadership Institute for middle and high school students from migrant and seasonal farmworker families since 1994. The academic yearlong leadership and educational access program uses popular education and cultural arts to build self-esteem, teach youth about social justice, and improve educational outcomes for youth from migrant and seasonal farmworker families in rural NC. Youth in the program meet for monthly, daylong meetings as well as quarterly overnight retreats, where they participate in workshops. As part of the program, youth also visit one college or university during the academic year. The program admits youth who work in farm work or who have a family member who works in farm work [17]. Participants are recruited through migrant education programs, high school counselors, and word of mouth. They can reapply for consecutive years.

The program takes place in Johnston County, an eastern NC county that has a population of approximately 182,000 residents, of whom 13% identify as Hispanic or Latinx [18]. The county has seen substantial increases in Latinx population over the last 20 years, and Spanish is spoken at home by 81% of the county’s Latinx population [18, 19]. High schools in the county have a Latinx dropout rate higher than the state and national average [19].

### Intervention

This partnership integrated technology (Apple iPad Air with unlimited AT&T cellular data plans) and training (health/information literacy) into the existing Levante Leadership Institute’s September 2017 to May 2018 cohort. ECU purchased the iPads and contracted for cellular data service during the program. The iPads were provided to the youth during the program orientation by program staff, and user agreements were established with the youth and their parents during the first overnight retreat of the program. Youth and parents signed the user agreements, which also mandated measures to protect devices from loss or theft. At the conclusion of the project, the iPads remained with the Levante Leadership Institute for future participants to use.

Information literacy training was provided in English in the context of a daylong college tour to ECU by an embedded public health liaison librarian. During the first half of this one-hour training, students engaged in a peer-to-peer discussion regarding the evaluation of online information. To initiate the discussion, students answered the following questions:

- Why should you think critically about information you find online?
- What would make you trust information you find online?
- What questions do you have about information you find online?

These questions sparked conversation around author credibility, timeliness of information, and bias.

Next, the students were introduced to the currency, relevance, authority, accuracy, and purpose (CRAAP) evaluation method [20] and discussed how each of the concepts related to online information. As a group, the students reviewed several online news stories through the online game “Factitious“ and tried to differentiate between accurate and false information utilizing elements of the CRAAP method. During this task, students engaged with the content and dove deeper into evaluating sources. Students discussed concepts such as publisher information, the article review process, and accuracy of claims made in the articles. After going through a fact-checking process, students had to reach a consensus on whether the article was “real” or “fake” and then test their decision in the game. This activity emphasized the importance of questioning online information and the necessity of reviewing content before making any decisions based on the information.

In the second half of the training, the group discussed the importance of evaluating information found online, especially if that information pertained to health. Students learned about the National Library of Medicine (NLM) resource MedlinePlus as an alternative to searching for online health information using a standard search engine. Different capabilities of the online resource were highlighted, such as the bilingual (English, Spanish) content and the availability of health education videos. At the end of the session, students were given time to look up various topics in MedlinePlus and ask any questions they had regarding the resource. Students were also made aware that MedlinePlus had been bookmarked on their iPads for future use.

### Data collection

The East Carolina University and Medical Center institutional review board approved the research protocol (#17-001303). After the students had used the iPads for approximately 3 months (and after parental consent, in English or Spanish, and student assent), author Mendez, who is trilingual, conducted semi-structured interviews individually with the students (n=10) in English between December 2017 and February 2018. All students who participated in the Levante Leadership Institute and used the provided iPads were eligible to participate in the interviews. Two students who were traveling at the time of the college tour and training were included because they had utilized the iPads.

The interviewer, who was a college student, had previously led activities with the students during the college tour, grew up in a Spanish- and Mayan language (Q’anjob’al)–speaking household in rural NC, was trained in qualitative data collection, and had previous interview experience. Interviews opened with a discussion of students’ experiences in the leadership program, followed by questions about using the iPads, and concluded with questions about what types of information they had sought with the iPads. (The full interview guide is available at the University of North Carolina Dataverse, DOI: http://dx.doi.org/10.15139/S3/IDWWVT.)

All interviews were digitally recorded. All recordings were transcribed using a smooth-verbatim protocol, in which stammers and “um”s were removed. Interviews lasted an average of 8.3 minutes (standard deviation [SD]=1.7, range: 6.0–11.3). The youth participants received a $25 gift card to Walmart at the completion of their interviews. Neither access to an iPad nor any other program benefits or activities were contingent on participating in the interviews.

### Analysis

Utilizing an inductive thematic approach, authors Mendez and Pories came to consensus on a codebook of key themes with input from two other authors (Cordova, Lee). Interview transcripts were then coded by one author using the codebook, and coding was discussed, modified by consensus, and finalized by the research team.

This research team brought multiple perspectives to the analysis process, because members held formal training in the disciplines of library science, public health, social work, and human development. The research team’s perspective included two analysts who were the first in their families to go to college, one fluent in Spanish and one proficient in Spanish (formerly fluent), one who studies the well-being of farming families, and one who had experience growing up in NC as part of an immigrant family from Central America. Two of the research team members identify as female and two as male. By triangulating the data using this diverse team and reaching consensus, the research team was able to control for bias [21] and ensure the credibility of the results. In sharing the results, we selected and presented representative themes from across the interview participants; however, we did not include participant numbers or aliases to eliminate the potential of deductive disclosure through triangulation.

Like systematic reviews and other study designs, researchers have identified core pieces of information that should be reported to ensure quality and transparency. Qualitative researchers are encouraged to utilize the thirty-two-item Consolidated Criteria for Reporting Qualitative Research checklist [22], which we have done.

## RESULTS

All ten participants in the leadership and college pipeline program participated in this project, which included having use of iPads, and in the interviews. All but two students in the program participated in the college tour and training. Participant characteristics are presented in Table 1. The cellular data plan, wireless connectivity, and iOS interface worked smoothly and were easy for participants to use. This finding was consistent across all participants and likely reflects the relatively strong cellular coverage of this area and the youths’ exposure to technology at home, at school, and through cellular phones. Challenges that program staff reported included managing access to Apple’s App Store, which required a credit card on file, and needing a staff member present to download new apps that students needed for school projects. The only challenges reported for ECU staff were dealing with the wireless provider’s billing errors.

**Table 1:**
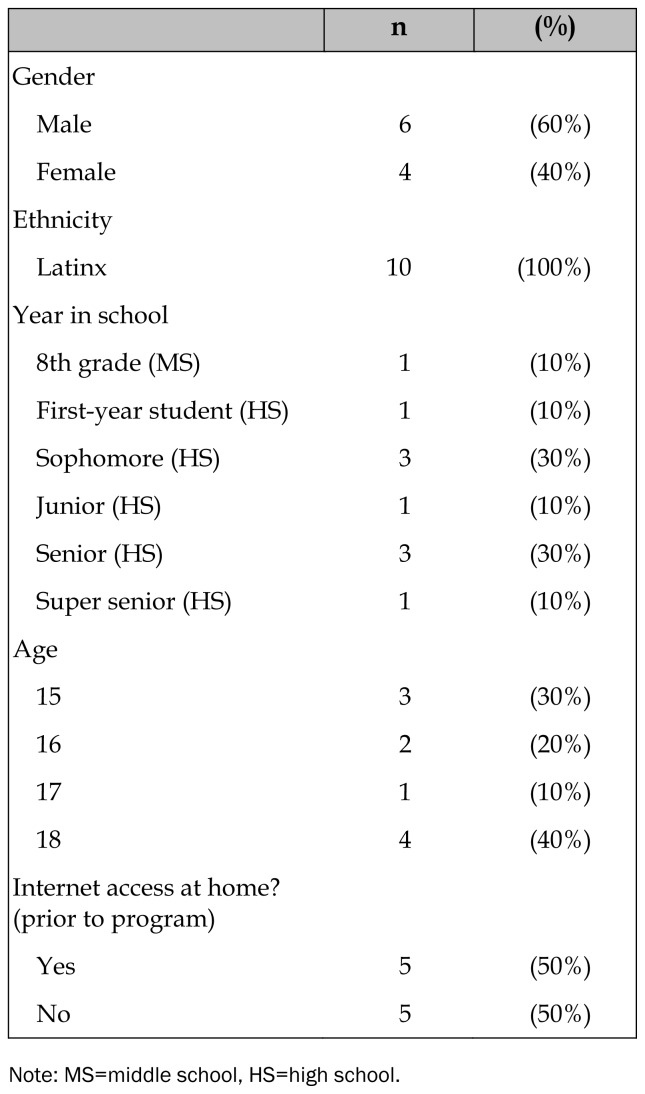
Participant demographics, n=10

	n	(%)
Gender		
Male	6	(60%)
Female	4	(40%)
Ethnicity		
Latinx	10	(100%)
Year in school		
8th grade (MS)	1	(10%)
First-year student (HS)	1	(10%)
Sophomore (HS)	3	(30%)
Junior (HS)	1	(10%)
Senior (HS)	3	(30%)
Super senior (HS)	1	(10%)
Age		
15	3	(30%)
16	2	(20%)
17	1	(10%)
18	4	(40%)
Internet access at home? (prior to program)		
Yes	5	(50%)
No	5	(50%)

Note: MS=middle school, HS=high school.

We identified four themes from the interviews with participants: (1) having access to the Internet can be transformative, (2) access resulted in increased knowledge of and interest in one’s own and others’ health, (3) “Google” is the norm, and (4) participant training increased self-efficacy to determine credible information sources and resources.

### Theme 1: Having access to the Internet can be transformative

The students reported that they enjoyed their experiences using the iPads. While half of the students indicated no prior experience with iPads, all of the students denied experiencing any challenges using the iPads, although one student indicated it was challenging to remember to charge the iPad. As one student summarized, “it’s easy to use” and “I think it was pretty cool.” Students reported no problems in connecting the iPads to the Internet, even though five students did not have Internet access at home prior to participating in this project.

All but one student volunteered that they used the iPads for school work and felt that they were helpful. Specifically, students reported that they used the iPads to research information for assignments or to do their homework, and one student reported allowing a family member to use the iPad to do their schoolwork as well. As one student noted, “It’s really hard to do your work on a phone and sometimes, the phone stops working or you don’t have any data or signal, and then you can’t turn in your work.” Another student noted the transformative power of having Internet access at home:

I feel like honestly, I get better grades. I made A’s because of the iPad, because…at the time, I didn’t have Internet at home. Since I had the iPad, I got to do things because at home—basically,…I wouldn’t have Internet, so I couldn’t work on my stuff. But since I got to have the iPad at home, I got to do stuff that I couldn’t do at school, which allowed me to perform better in school, and I feel like it really helped me a lot.

### Theme 2: Access resulted in increased knowledge of and interest in one’s own and others’ health

Students reported that they searched for information about health using their iPads. All but three students reported looking for health information for themselves, their families, or their friends using the iPads. One student noted, “it kind of helps you to just go, just kind-of like a general idea of what you might have.” Another student indicated, “my grandma and the rest of my maternal family side, they have diabetes. So, she would ask me what would be something good to take to lower the blood sugar or to make it go higher.”

The students reported that they looked up health information for themselves, not only to identify underlying problems from symptoms they were experiencing, but also to see how to deal with mental health issues (e.g., depression) or to learn more about issues such as nutrition. They further utilized the iPads to search for information for family and friends on health topics including cancer, bipolar disorder, miscarriages, diabetes, hydration during hot weather, skin care, and nutrition. They also reported using the Internet for information related to politics, cars, and directions to various places. Two students noted searching about symptoms that led directly to seeking medical treatment for problems. One stated:

I have like a little red dot, and it turned out to be ringworm, and I had to get it checked. I searched it up, and then it turned out I had to go to the doctor and get it checked, but I had to figure out what it was.

Another reported:

My dad had a cold two or three weeks ago, so we were searching it up, and it just said that he had a cold. But it was kind of like some of the symptoms were the same, but then he had other ones. So, he ended up going to the hospital because it was really bad, but we could kind of get a sense of what it was.

Several students indicated that being able to search for health information on the iPads was useful. One student stated, “it was helpful because, again, my family was more informed, and they knew how to treat it better.” Indeed, one of the students indicated the reach of this health information was not limited to just their families because family members “would give their co-workers advice.”

### Theme 3: “Google” is the norm

Regarding how they searched for information, all of the students reporting using Google to search for information: “I go on to Google, I just type whatever I want to search” was the overall sentiment they expressed. Or, as aptly stated by one, “I just put it in Google, and then I would find things.” As noted by another, Google led to “trusted sources that relates to the topic that I’m looking for,” which was what the students stated they were interested in. Students indicated that they wanted a search engine that was easy to use, was organized, and allowed them to find information specific to what they were searching for. Despite our training, reported use of MedlinePlus was limited to a single student during our follow-up interviews.

### Theme 4: Participant training increased self-efficacy to determine credible sources and resources

During the interviews, which occurred two to four months after our training, students were aware that, as one simply stated, “you shouldn’t trust…all websites because they might have…fake information.” As one student noted, “There’s things that aren’t trustworthy. Some of it’s not real. It’s all very exaggerating and then there’s ones that just tell you how it is.” Students commented that the training they received helped them develop skills to better differentiate fake news from real news. One student noted:

Because we all know that the Internet sometimes is not true all the time, but see, looking at the little things like EDU, or like .org, or actual websites where the information might be right, because a lot of people just search up, and they put blogs. They find blogs and then put it there, and that’s not the right information. But if you kind of know that the good information comes from the EDU, or org, or some university, then they kind of figure out that that might be a good website for them to look at.

## DISCUSSION

### Principal findings

Our most striking finding was the transformative power of having access to the Internet on health information and education. Our participants clearly articulated that having Internet at home through the iPads made their educational experiences better and allowed easier access to health information. Provision of iPads and Internet connectivity to middle and high school age youth from farmworker families in the context of a leadership and college pipeline program was possible and well received.

Additionally, our interactive training on source credibility was remembered and influential at the time of the interviews. Yet, we had no success in increasing use of MedlinePlus. It also remains unclear if we increased participants’ self-efficacy for identifying high-quality sources beyond their actual skills. That is, we have some concern that our training raised participants’ perception of being able to identify high-quality health information substantially more than might be warranted by a single training. Our findings suggested the importance of programmatic efforts to ensure access to health information through Internet access, while continuing to build and strengthen educational opportunities regarding information literacy.

### Findings in context

Our findings fit in the broader literature on substantial inequalities in health and justice for farmworkers and their families compared to the general population [1, 23], including in access to basic utilities and housing [24]. Our findings affirm that access to the Internet is important for health and education [25, 26]. Our results support the idea that health information literacy and information access interventions can be part of broader efforts to address these inequalities [2].

Finally, our results have implications for both public health practice and libraries. Our results fit with existing literature about the important overlap in competencies and skills between health promotion practitioners and health librarians [27]. Regarding training and education, we noted that the most interactive portion of our training proved to be the part that was most remembered and utilized. This is consistent with prior research showing the importance of active learning in library instruction for high school students [28].

Our results show the value in connecting health and leadership programs with libraries both for information literacy training and for knowledge about consumer health information resources. It is important to integrate information literacy with efforts to improve access to information. We see a potential role for medical libraries in partnering with community organizations, community college libraries, and public libraries to increase health information literacy and utilization of reliable consumer health resources. We also see a potential synergy between farmworker advocacy and service organizations and public libraries in increasing Internet access for youth from farmworker families. Public libraries and community organizations should consider developing relationships to increase information literacy and improve access to the Internet.

### Limitations

There are important limitations to this research. The limited geographic reach of our program, which was conducted in a single region of NC, prevents us from drawing conclusions for other regions of the state or the country. This is especially true in areas of the country where even cellular Internet service is not available. Our program was implemented in the context of a single nonprofit organization’s existing programming, and the unique strengths of that nonprofit might have influenced our findings. The qualitative approach prevents us from quantifying impact, and future work should consider including quantitative measures. Additionally, a one-hour information literacy session would be unlikely to make substantial changes in behaviors (although it did appear to impact the way participants thought about information). Our interviews were short but were appropriate for our evaluation purposes and for the age of our participants. Due to program time constraints, we were unable to complete member checks where participants reviewed their transcripts and commented upon our themes. However, we developed the themes using an interdisciplinary research team and received peer feedback by presenting preliminary results to migrant outreach workers and other researchers. Future research should consider higher-impact strategies to improve use of reliable consumer health information sources from NLM.

## CONCLUSION

We hope this work calls attention to gaps in Internet access that should be considered in leadership development, health, and library efforts that are designed to address health inequalities that migrant and seasonal farmworkers and their families face. Gaining access to the Internet at home has the potential to be one critically important component in addressing health and educational inequalities among farmworker families. Efforts to improve health literacy among the families of migrant and seasonal farmworkers should consider increasing access to the Internet in combination with interactive health information literacy training. The transformational power of being able to access the Internet at home is an important area of intervention and should be considered by funding agencies and program developers.
